# Assessing Osteopathic Medical Students' Knowledge, Attitude, and Perspective on the Use of Assisted Reproductive Technologies (ART)

**DOI:** 10.7759/cureus.83909

**Published:** 2025-05-11

**Authors:** Anishaa Patel, Melissa M Capote, Angellar Manguvo, Benford Mafuvadze

**Affiliations:** 1 Anatomy and Molecular Medicine, Alabama College of Osteopathic Medicine, Dothan, USA; 2 Medicine, Alabama College of Osteopathic Medicine, Dothan, USA; 3 Graduate Health Professions in Medicine, University of Missouri-Kansas City, Kansas City, USA

**Keywords:** assisted reproductive technologies, childbearing, fertility, fertility challenges, medical students

## Abstract

Background: Medical students face unique challenges in balancing training and family planning, often delaying childbearing due to the rigorous demands of the medical curriculum. In general, female physicians have their first childbirth at an age higher than that of women in the general population, increasing their risk of fertility challenges. Despite awareness of these challenges, medical students often prioritize professional commitments hoping to rely on assisted reproductive technologies (ART) later. However, ART's success rates decline with age, and its success in older women often depends on the use of eggs harvested early and stored. A comprehensive understanding of ART will help medical students make informed decisions on childbearing. This study evaluated medical students' knowledge of ART and assessed their attitudes toward its use.

Methods: This study employed a survey research design to evaluate medical students’ knowledge, awareness, and attitude toward ART. The custom-designed instrument was developed based on a review of relevant literature, informal interviews, and expert consultations and adapted from an earlier survey tool. The final questionnaire included three sections: nine demographic items, 15 knowledge items assessing understanding of various topics related to fertility and ART, and 17 attitude items evaluating views on various issues pertaining to ART. The survey also included open-ended text boxes for qualitative responses. Data were collected using an electronic survey administered through Qualtrics (Qualtrics International Inc., Seattle, WA, US) over eight weeks. Collected data were analyzed using SPSS version 30 (IBM Corp., Armonk, NY, US). Descriptive statistics summarized demographic characteristics, knowledge, and attitudes. Independent t-tests, ANOVA, and chi-squared statistics assessed differences across demographic categories. Thematic analysis of open-ended responses identified emerging themes.

Results: The study involved 113 medical students. The majority were first-year female students aged 25 years and older. Most participants (n = 106, 93%) did not have children but planned to have children in the future, particularly after residency. While participants demonstrated strong knowledge of ART, especially regarding the impact of age on fertility, most had an average understanding of birth control effects and the male partner’s age on pregnancy outcomes. Knowledge about financial aspects, health risks, and long-term outcomes of ART-conceived children was comparatively lower. Attitudes toward ART were generally favorable, with strong support for egg and sperm donation and moderate interest in egg harvesting and freezing. There was considerable support for making in vitro fertilization (IVF) available to single women and same-sex couples, but a preference for limited federal funding for IVF and a moderate willingness to pay for it. In general, participants opposed the use of surrogate mothers by couples capable of carrying pregnancies and had divided views on the designation of frozen embryos as “children.”

Conclusions: Overall, this study revealed limited knowledge about ART among medical students, particularly regarding the financial and long-term implications of using ART. These results highlight the need for earlier integration of fertility and ART education into medical school curricula, which would better prepare students to make informed reproductive decisions.

## Introduction

The time it takes to complete a medical degree in the United States from the beginning of the undergraduate program to completion of residency training is on average 11 years. It may take even longer for non-traditional students such as those who did not major in science during undergraduate programs or those who were involved in other careers prior to switching to study medicine. The rigorous and demanding nature of medical training often compels students to postpone significant life events, such as marriage and childbearing, until they have completed residency training [1,2]. This postponement is reflected in the 34-35-year average age of first childbirth among female physicians in the United States, which is substantially higher than the general population’s average of 29 years [3]. Such delays in childbearing place female physicians at an elevated risk of facing fertility challenges, as age-related fertility decline becomes a significant barrier by the time they pursue biological parenthood [3,4]. Notably, research has shown that 25% of female physicians attempting conception report experiencing infertility [4]. Furthermore, other studies have revealed that female physicians experience significantly higher rates of infertility and unintentional childlessness compared to the general population [3,5]. This issue is compounded by the findings that 28% of these physicians facing fertility challenges expressed regret for not attempting conception earlier [1]. The prioritization of medical careers over childbearing not only affects physicians’ personal lives but also can strain familial relationships, as some report tension with significant others over differing priorities [6,7]. Moreover, the desire for childbearing and family-building often influences career decisions, with many female physicians selecting residency programs perceived as more accommodating to family life. This trend highlights systemic barriers preventing women from equally pursuing both their desired careers and family goals [5]. Collectively, the findings discussed above underscore the multifaceted challenges faced by female physicians as they navigate the complexities of balancing familial responsibilities and professional aspirations.

Despite being aware of the challenges associated with delayed childbearing, many medical students continue to prioritize their professional commitments, with the hope of resorting to assisted reproductive technologies (ART) in the event of facing fertility difficulties later in life [1]. This reliance on ART is partly shaped by media representations of celebrities giving birth in their 50s, which propagates the perception that ART can effectively counteract reproductive aging [8]. However, this optimism frequently overlooks the inherent limitations of ART, particularly its declining success rates with advancing maternal age [8]. While, indeed, ART has made substantial advancements over the past four decades, its highest success rates are observed in women under the age of 35. By the mid-40s, even with ART, the probability of conception diminishes significantly. For women over 45, success often hinges on proactive measures such as early egg harvesting and cryopreservation [8]. In the absence of such interventions, the window for successful ART becomes increasingly restricted [8].

In addition to the challenges posed by aging, another significant barrier is the prohibitive cost of ART, which often requires multiple cycles to achieve success, particularly for women older than 40 [9]. Compounding this issue, ART is rarely fully covered by health insurance, leaving patients to bear substantial out-of-pocket expenses [10]. Financial barriers are further exacerbated by sociopolitical shifts that increasingly influence the accessibility of family planning resources for women. Recent legislative changes in many states, including Alabama, have raised concerns about the future availability of specific ART options. We think that this will result in female trainees making critical decisions about childbearing without assurance of access to these technologies.

Cumulatively, the challenges outlined above highlight the critical need to equip medical students with accurate and comprehensive knowledge to make informed decisions about their reproductive health and family planning. Previous research has, however, highlighted a general lack of adequate knowledge about various aspects of fertility, including ART [11,12]. Unsurprisingly, many female physicians report regret for not attempting conception earlier when confronted with fertility challenges later in life [1]. 

The primary objective of this study was, therefore, to evaluate the general knowledge of medical students regarding ART and to assess their attitudes toward its use. While most prior studies have focused on the general public, including undergraduate students, these populations differ significantly from medical students, who face unique age-related fertility challenges when they try to conceive. We hypothesized that medical students would overestimate the success rate of ART at older ages. Our study specifically examined the attitudes and knowledge of ART among students at an osteopathic medical college. It is imperative that medical students develop a comprehensive understanding of their reproductive options, including the risks and benefits of delaying childbearing, to make well-informed decisions about their family planning in the context of their demanding medical training and future careers [13].

## Materials and methods

This study employed a survey research design to evaluate medical students’ knowledge, awareness, and attitude toward ART.

Instrumentation 

Several survey items were adapted from an instrument developed by Szalma and Bitó [9], with additional items incorporated to ensure comprehensive coverage of core components of ART and alignment with the specific objectives of the present study. The adaptation process was informed by a review of relevant literature, informal interviews with students, and consultations with subject-matter experts. We then conducted a pilot study to evaluate the instrument’s clarity, relevance, and comprehensiveness; feedback from this phase guided subsequent refinements to enhance both validity and reliability. The final version of the questionnaire comprised three distinct sections, as detailed below.

Demographics

The section gathered information on participants’ gender, year of study, ethnicity, religion, sexual orientation, and age, which provided a comprehensive profile of the participants for further analysis.

Knowledge

The knowledge subscale included 15 items assessing participants’ knowledge and understanding of fertility issues and other aspects of ART. Participants rated the accuracy of statements on topics such as the effects of long-term birth control, in vitro fertilization (IVF) success rates and costs, age-related impacts on fertility, benefits of egg freezing, and the influence of male partner age. Additionally, they were evaluated on awareness of IVF-related health risks, long-term outcomes of ART, and the impact of factors like alcohol consumption and hot baths on fertility. Cronbach’s alpha coefficients were computed to evaluate the reliability and internal consistency of the knowledge subscale. Consistent with findings reported by Szalma and Bitó [9], from whom the original scale was adapted, the 15-item knowledge subscale demonstrated moderate internal consistency (α = 0.472), which is slightly higher than the value reported in the original study (α = 0.408).

Attitudes

The attitudes subscale collected participants’ views on sperm and egg donation, egg freezing, and surrogate motherhood. It also assessed opinions on using IVF for initial or subsequent children, delaying parenthood, and managing compromised fertility. Additionally, the subscale explored attitudes toward government funding for IVF, out-of-pocket costs, access for single women and same-sex couples, and the legal status of frozen embryos. Open-ended text boxes were included, enabling participants to provide qualitative responses and offer further insights into their attitudes.

Sampling and data collection procedures

Data were collected from students at Alabama College of Osteopathic Medicine. Following approval of the study by the Institutional Review Board (IRB# 24-02-26-001), the survey was administered electronically via Qualtrics (Qualtrics International Inc., Seattle, WA, US) and remained open for eight weeks, from March 1, 2024, through April 26, 2024. The study population comprised current medical students (OMS I-IV) enrolled at the Alabama College of Osteopathic Medicine (ACOM), with a total enrollment of 751 students. In the recruitment letter, students who had completed or were currently enrolled in the Obstetrics and Gynecology course were asked to refrain from participating in order to maintain comparability in baseline knowledge across participants. Participation in the study was voluntary, and electronic informed consent was obtained from all participants prior to the completion of the survey.

Data analysis

Data were extracted from Qualtrics and imported into SPSS version 30 (IBM Corp., Armonk, NY, US) for analyses. Due to the limited representation in certain demographic categories, we consolidated some groups into broader categories to allow meaningful statistical comparisons while addressing issues of small sample sizes within subgroups. This involved creating binary variables; for example, ethnicity was recoded into Caucasian/White and non-White.

All 15 items on the knowledge subscale were systematically coded on the 0-4 Likert scale, where higher values corresponded to more accurate responses. Similarly, Likert-scaled items in the attitude subscale were re-coded on a 0-4 scale, where higher scores indicated more positive attitudes and lower scores reflected less favorable perspectives. Categorical responses in the attitude subscale were retained in their original form for analysis.

Following data cleaning and recoding, descriptive statistics were calculated to summarize respondents’ demographic characteristics as well as their knowledge and attitudes on ART. Independent t-tests and ANOVA were employed to assess differences in knowledge and attitude mean scores across various demographic categories, while chi-squared statistics were used to analyze associations among categorical variables.

Thematic analysis was conducted to examine the open-ended responses, using an inductive approach to identify emerging patterns and themes. Two authors independently coded the data and subsequently met to review and refine the themes collaboratively, thereby enhancing the consistency of the analysis. Responses were organized into thematic categories, and illustrative verbatim quotes were included to substantiate the findings and provide a richer understanding of participants’ perspectives.

## Results

A total of 133 medical students initially participated in the study. Following data cleaning procedures, which involved the removal of incomplete or ineligible responses, the final analytic sample comprised 113 participants, yielding an overall response rate of approximately 15%. Female participants comprised the majority of the respondents. Most participants were in their first year of study, with a significant portion identifying as White. Similarly, a larger number of participants identified as Christian. The majority of respondents were heterosexual, and the predominant age group was 25 years and older. Table 1 provides further details of the demographic distribution of the sample.

**Table 1:** Demographic characteristics of participants

	Female N (%)	Male (%)	Not given (%)	All (%)
Year of study				
Year 1	36 (49)	20 (56)	1 (0.9)	57 (50)
Year 2	19 (26)	5 (14)	1 (0.9)	25 (22)
Year 3	9 (12)	5 (14)	1 (0.9)	15 (13)
Year 4	10 (14)	6 (17)	0 (0)	16 (14)
Ethnicity				
Caucasian/White	42 (57)	30 (83)	2 (2)	74 (65)
Non-White	32 (43)	6 (17)	1 (0.9)	39 (35)
Religion				
Christianity	49 (66)	21 (58)	1 (0.9)	71 (63)
Non-Christian	25 (34)	15 (42)	2 (2)	42 (37)
Sexual orientation				
Heterosexual	64 (86)	33 (92)	1 (0.9)	98 (87)
Non-heterosexual	10 (14)	3 (8)	2 (2)	15 (13)
Age				
16-24	15 (20)	4 (11)	1 (0.9)	20 (18)
25 and older	59 (80)	32 (89)	2 (2)	93 (82)
Total	74 (65)	36 (32)	3 (3)	113 (100)

Regarding current and future childbearing plans, most participants did not have children at the time of the survey. However, the majority indicated intentions to have children in the future, with a notable proportion planning to do so after completing their residency. These results are summarized in Table 2. No statistically significant differences were observed between male and female participants for both current and future childbearing plans (χ^2^ (5, n = 113, p > 0.05)). 

**Table 2:** Current and future childbearing plans for participants

	Female N (%)	Male N (%)	Not given	All N (%)
Have children				
Yes	3 (4)	3 (8)	1 (0.9)	7 (6)
No	71 (96)	33 (92)	2 (2)	106 (93)
Plan to have children				
During med school	9 (12)	9 (25)	1 (0.9)	19 (17)
During residency	6 (8)	7 (19)	1 (0.9)	14 (12)
After residency	16 (22)	8 (22)	0 (0)	24 (21)
Not now, yes in the future	36 (49)	10 (28)	1 (0.9)	47 (41)
No plan to have children	6 (8)	2 (6)	0 (0)	8 (7)
Not given	1 (0.9)	0 (0)	0 (0)	1 (0.9)
Total	74 (65)	36 (33)	3 (3)	113 (100)

Knowledge and understanding of ART

The 15 items measuring knowledge of ART-related issues were averaged to compute each participant’s overall knowledge score. Results indicated a relatively strong understanding, particularly regarding the impact of age on fertility. Awareness of topics such as the effects of birth control on fertility and the influence of the male partner’s age on pregnancy outcomes showed average levels of understanding. However, knowledge about the financial aspects of IVF, associated health risks, and long-term health outcomes of ART-conceived children received comparatively lower mean scores. Table 3 details the average scores for knowledge about ART.

**Table 3:** Participants’ average scores of knowledge about ART ART: assisted reproductive technologies; IVF: in vitro fertilization

ART knowledge item	Mean	SD
Taking birth control pills for >5 years negatively affects a woman’s fertility	2.63	1.13
Over 40 years old, the success rate of fertility among women is around 50%	2.79	0.87
The total cost for one cycle of IVF is under $5,000	3.06	0.84
There is a progressive decrease in a woman’s ability to become pregnant at >35 yrs	3.3	0.87
The rate of miscarriages is significantly higher for women in their 40s than for women in their 30s, even for physically fit women in excellent health	3.19	0.87
Egg-freezing before the age of 35 can significantly prolong a woman’s fertility	2.68	0.91
The age of the male partner is an important factor in the chances of becoming pregnant	2.26	1.36
The use of IVF poses health risks for a woman	2.51	0.99
Children conceived using ART have more long-term health problems than children conceived without the use of these fertility treatments	1.21	0.81
Most fertility conditions are caused by problems with the woman’s fertility	2.72	0.90
Most women have to go through IVF more than once to have a baby	2.84	0.86
The upper age limit for a man to be treated at most fertility clinics in the US is 55	2.05	0.79
Excessive alcohol consumption can increase infertility among women and men	2.62	1.41
Frequent use of hot baths and/or saunas can damage sperm	2.52	1.16

Attitudes toward ART

For the categorical items on the attitude subscale, frequency distributions indicated strong support among respondents for practices such as egg and sperm donation. Contrary to this positivity, fewer participants expressed comfort with the idea of their partner becoming a sperm or egg donor. Interest in egg harvesting and freezing was moderate among the respondents. Table 4 shows specific frequency percentage distributions for attitude toward ART for the categorical items. Opinions on whether ART encourages delayed childbearing were mixed. There was considerable support for making IVF available to single women and same-sex couples.

**Table 4:** Consideration for the use of IVF treatment among respondents IVF: in vitro fertilization; ART: assisted reproductive technologies

Opinion item	Yes N (%)	No N (%)	Neutral N (%)
Do you think IVF and other ART encourage delayed childbearing?	34 (30)	43 (38)	36 (32)
Do you support the practice of egg donation?	80 (71)	15 (13)	18 (16)
Do you support the practice of sperm donation?	81 (72)	14 (12)	18 (16)
Would you or your partner consider harvesting and freezing eggs?	66 (58)	25 (22)	22 (20)
Do you think that IVF should be available to single women?	74 (66)	21 (19)	17 (15)
Do you think that IVF treatment should be available to same-sex couples?	79 (70)	18 (16)	15 (13)
Would you be comfortable with your partner being a sperm or egg donor?	32 (28)	56 (50)	24 (21)

The remaining five Likert-scaled items measuring attitudes toward ART, which were coded on a 0-4 Likert scale, were averaged to compute each participant’s overall attitude score. Results showed that participants generally held favorable views toward the use of ART. However, when asked about the number of IVF rounds the federal government should fund, responses suggested a preference for limited funding. Opinions on paying for IVF showed moderate willingness. There was strong opposition to the use of surrogate mothers by couples capable of carrying their own pregnancies. Finally, views were divided on whether frozen embryos should be considered “children” following the recent Alabama Supreme Court ruling [14]. Table 5 details the mean scores for Likert-scaled attitude items.

**Table 5:** Average scores of attitudes about ART IVF: in vitro fertilization; ART: assisted reproductive technologies

ART attitude item	Mean	SD
What do you think about the use of ART in humans?	3.22	1.02
What do you think should be the maximum number of rounds of IVF that the federal government should fund for any individual/couple?	2.27	1.30
How much would you pay to have a child through IVF?	2.93	1.44
What do you think about the use of surrogate mothers by couples who are capable of carrying their own pregnancy?	1.62	1.50
In line with the recent Alabama Supreme Court Ruling, do you think frozen embryos should be considered as "children"?	2.09	1.60

Further analysis of ART knowledge and Likert-scaled attitude items indicated no significant differences across most demographic variables, such as year of study, ethnicity, and religion. The sole notable difference was in gender, where male participants exhibited slightly higher attitude scores compared to female participants. Table 6 presents a summary of the mean scores and demographic variations in knowledge and attitudes toward ART.

**Table 6:** Demographic differences in attitudes and knowledge of ART Independent sample t-test (df = 111) and one-way ANOVA (df = 3) were used to analyze various demographic factors and how they influenced student attitudes and knowledge of ART. *p < 0.05 was considered statistically significant. ART: assisted reproductive technologies

	N (113)	Mean score	SD	Test statistic for attitudes	Mean score	SD	Test statistic for knowledge
Gender				t = 1.589, p = 0.115			t = 2.476, p = 0.015*
Female	74 (65)	2.03	0.91		2.57	0.33	
Male	36 (32)	2.32	0.83		2.74	0.38	
Not given	3 (3)	N/A	N/A				
Year of study				F = 1.816, p = 149			F = 1.991, p = 120
Year 1	57 (50)	2.19	0.84		2.56	0.33	
Year 2	25 (22)	1.88	1.09		2.61	0.36	
Year 3	15 (13)	2.00	0.93		2.66	0.34	
Year 4	16 (14)	2.50	0.56		2.80	0.39	
Ethnicity				t = 0.019, p = 0.985			t = 2.200, p = 0.030*
Caucasian/White	74 (65)	2.14	0.97		2.67	0.38	
Non-White	39 (35)	2.15	0.73		2.52	0.29	
Religion				t = 1.888, p = 0.062			t = 0.275, p = 0.784
Christianity	71 (63)	2.26	0.87		2.62	0.36	
Non-Christian	42 (37)	1.93	0.89		2.60	0.35	
Sexual orientation				t = 207, p = 0.836			t = 0.0497, p = 0.620
Heterosexual	98 (87)	2.15	0.90		2.61	0.37	
Non-hetero.	15 (13)	2.10	0.85		2.66	0.23	
Age				t = 0.071, p = 0.943			t = 1.158, p = 0.249
16-24	20 (18)	2.13	1.16		2.53	0.29	
25 and older	93 (82)	2.15	0.83		2.63	0.36	
Grand total	113 (100)	2.14	0.89		2.62	0.33	

Opinions regarding the role of the federal government 

Further analysis of the role of the federal government in ART revealed varied opinions depending on the situation. For couples unable to conceive their first child, views were divided between federal and private funding, with a small minority opposed. However, most preferred the use of private funding for additional children. For those delaying childbearing due to professional or national commitments, federal support was most preferred. When fertility was compromised by medical conditions like cancer, federal funding was favored by the majority. Table 7 details these perspectives, highlighting differences based on specific contexts.

**Table 7:** Overview of opinions on appropriate funding of ART across various situations ART: assisted reproductive technologies; IVF: in vitro fertilization

Under which circumstances do you think IVF should be funded by the federal government or by individuals	Federal government	Privately	Should not be available
When having a first child and unable to conceive naturally	51 (45)	56 (50)	6 (5)
When having another child after having at least one but unable to conceive naturally	24 (21)	82 (73)	7 (6)
When a decision has been made to have a child later in life due to medical studies and other national commitments such as military deployments	59 (52)	48 (43)	6 (5)
When fertility has been risked/sacrificed (e.g., due to cancer)	57 (50)	49 (44)	7 (6)

Open-ended responses regarding ART reflected a broad spectrum of opinions. Key themes included opposition to government involvement, ethical concerns, and the physical toll of IVF treatments.

Opposition to Government Involvement

Respondents who strongly opposed government interference had comments like “the federal government shouldn’t be involved at all” and “government doesn’t belong in healthcare and should certainly not be funding people’s reproduction.”

Ethical Concerns

Ethical objections were also raised in the open-ended responses, particularly around financial responsibility, with one respondent noting, “It is not right to force taxpayers to pay for individuals’ fertility treatment.” Faith-based and moral concerns emerged as well, with another calling the practice “an abomination.”

Physical Toll of IVF Treatments

Other participants expressed concerns about the physical burden of IVF on mothers. One respondent stated, “IVF fertility treatments (for egg harvesting, etc.) are physically challenging on prospective mothers, and such repeated procedures with little physiologic benefit are difficult to justify.” Others highlighted the unpredictable nature of IVF, emphasizing that “it can take many cycles for a couple to conceive successfully” and pointing out the potential for multiple births in a single cycle.

## Discussion

In recent years, there has been growing interest in evaluating the understanding of fertility-related issues, including ART [9,15-19]. Building on this trend, our study assessed the knowledge, attitudes, and perspectives of medical students at an osteopathic medical school regarding the use of ART. 

The majority of participants in this study were female, reflecting broader societal trends where fertility and childbearing topics tend to generate greater interest among women than men [2]. Most of these students indicated plans to have children after completing residency, a time when the majority will be older than 32 years. This finding is particularly significant given that current evidence shows female fertility begins to decline at age 32 and decreases sharply after age 37 [2].

Our study demonstrated a generally strong understanding among students regarding the impact of female age on pregnancy outcomes. The success of IVF at older maternal ages is highly dependent on using eggs harvested at a younger age [13]. As awareness of age-related fertility decline grows, egg harvesting and freezing have become increasingly relevant topics of discussion among female medical students and resident physicians [13]. Reflecting this trend, a significant majority of participants in this study expressed a willingness to consider harvesting and storing their own eggs, provided financial support is available. This highlights the growing recognition of fertility preservation as an option. 

Previous studies of university students have consistently documented a tendency to overestimate their understanding of fertility-related topics [11,12,20,21], despite an actual general lack of knowledge about key aspects. Notably, these studies identified significant knowledge gaps, particularly regarding the maternal health risks associated with ART and the long-term health outcomes of ART-conceived children. Strikingly, our findings revealed similar trends among participants, which contradicted our initial hypothesis that medical students, irrespective of their stage of training, would possess a higher baseline knowledge of these topics compared to the general population [2]. 

Our study highlighted a general lack of understanding among participants regarding the impact of male partner age on fertility. While fertility discussions often focus on women, comparatively little attention is given to the decline in male fertility, despite evidence indicating that both sperm quality and semen parameters deteriorate with age [2]. Another area of limited knowledge was the health outcomes of children conceived through ART. Participants also demonstrated insufficient knowledge about the financial aspects of ART procedures, such as IVF. Many were unaware that ART often requires multiple cycles, with costs escalating significantly with each attempt. 

The lack of knowledge displayed may, in part, be explained by the fact that approximately half of our participants were first-year medical students who had not yet taken courses in endocrinology or reproductive health, subjects traditionally introduced in the second year of medical education. Additionally, variations in premedical education across states could contribute to these knowledge gaps. Some states impose significant legal restrictions on the discussion of ART and related topics, leading to inconsistencies in the depth and scope of reproductive health education [2]. Moreover, current medical curricula may also contribute to these gaps, as in-depth coverage of ART is often considered beyond the scope of preclinical education in most medical programs. 

We also evaluated participants’ attitudes toward the use of ART and related techniques for conception. Overall, our findings revealed that participants held a positive attitude, particularly regarding egg and sperm donation. Interestingly, while they were generally supportive of these practices, participants expressed greater hesitation about their own partners serving as egg or sperm donors. Previous studies have similarly noted reluctance to donate gametes, often attributed to concerns about potential binding legal obligations that may arise in the future or fears that the donor might develop an emotional bond with the conceived child [22]. 

Participants’ views on federal government funding for ART were mixed, with stronger support observed in cases involving couples attempting to conceive their first child, delays due to national service or professional training, or when fertility is compromised by medical conditions. There was also significant support for making IVF accessible to single women and same-sex couples, reflecting an overall embrace of diversity among medical students. However, most participants expressed strong opposition to the idea of couples capable of carrying their own children utilizing surrogate mothers. This opposition aligns with bioethical concerns frequently raised regarding surrogacy, including issues surrounding the autonomy of the surrogate mother, as well as the potential exploitation and commodification of both the surrogate mother and the child [23,24]. 

Religion usually plays a significant role in shaping perspectives on bioethical issues, with varying viewpoints among different religious groups regarding ART. For instance, the Catholic Church opposes ART, while Protestant and other non-denominational Christian groups generally permit its use among their members [22]. Judaism allows all forms of ART, provided the oocyte and sperm come from a married couple [22]. Within Islam, Sunni Muslims may use ART if the couple is married, whereas Shia Muslims can also utilize third-party donations under specific conditions [22]. Given these diverse religious positions, we hypothesized that religion would significantly influence participants’ attitudes toward ART. However, contrary to our expectations, most participants reported that religion was not a major factor in shaping their attitudes or perspectives on ART. 

The qualitative findings from this study underscore the complexity and diversity of perspectives surrounding ART, particularly in the areas of government involvement, ethical considerations, and the physical and emotional demands of fertility treatments. These findings suggest a deep interplay between personal values, societal norms, and practical challenges in shaping attitudes toward ART. Opposition to government involvement highlights a broader tension in public discourse about the role of state funding in healthcare, particularly when addressing issues perceived as private or elective, such as fertility treatments. Meanwhile, the ethical concerns raised illustrate the moral and societal complexities inherent in ART. 

The limited knowledge observed among participants in this study may stem from the insufficient emphasis on reproductive and family planning education within the US educational system prior to medical school [13,25]. This study’s findings also align with previous researchers underscoring the need to incorporate fertility and ART-related topics earlier in the medical curriculum. Educating medical students on fertility decline and available options, such as oocyte preservation, can significantly enhance their understanding and preparedness for future reproductive planning [2]. Given the increasing number of female students entering medical training over the past few decades, the need for this education has become more pressing [26,27]. Previous studies and calls to action have emphasized the integration of family planning and fertility education in residency programs [28]. While such initiatives are commendable, the timing of such interventions should be reconsidered. Fertility education and support should be offered earlier, during medical school, as critical decisions about family planning and career progression are often made prior to residency. In recent years, some graduate medical education (GME) programs have expanded insurance coverage and financial assistance for egg harvesting and other fertility care options [28]. While these initiatives are plausible, offering similar provisions to medical students earlier in their training could empower them to make proactive, informed decisions and engage in advanced planning. This approach has the potential to reduce career-related disruptions and minimize future regrets associated with delayed family planning [7].

Limitations

This study was limited by the small sample size, which made it difficult for us to compute in-depth dimensional comparisons across different demographic groups. Female, Caucasian, and first-year students were overrepresented compared to the population of the osteopathic medical school. The study was also limited to one osteopathic medical school and further limited by convenience sampling due to its voluntary nature. It is most likely that it drew more attention from students interested in the subject, which might have biased some of our analysis on knowledge and attitudes. A study involving multiple medical schools, especially in different geographic regions, would allow for more detailed comparison among students especially as sociopolitical views are increasingly regional in the United States. Interpretation of findings from this study should be done cautiously, taking the proper context into perspective. Despite the stated limitations, most of our findings concurred with findings from previous studies conducted with university and medical students.

## Conclusions

This study, in agreement with several studies, confirms a need for educating and making medical students aware of challenges associated with the use of ART. This study unearthed some knowledge gaps in ART-related topics among medical students, particularly regarding the financial and long-term implications of using ART. These results highlight the need for earlier and more comprehensive integration of fertility and ART education into medical school curricula. Addressing these gaps may better prepare students to make informed reproductive decisions. The study also calls for the provision of funding to medical students for coverage of ART-related procedures like egg harvesting and storage.

## Appendices

Survey instrument

We would like to invite you to participate in this study entitled “Assessing Osteopathic Medical Students' Knowledge, Attitude, and Perspective on the Use of Assisted Reproductive Technologies (ART).” A significant proportion of medical students delay childbearing until they are done with medical school. The main aim of this research is to measure the knowledge and attitudes of medical students to fertility and the use of assisted reproductive technologies (ART).

The questionnaire is completely anonymous and takes only 5-10 minutes to complete. Your name is not required, and no one can identify individual answers in this study. You have the right to terminate the survey at any time prior to submission. If you complete and submit this survey, it will be assumed that you have consented to participate in the study. When you have completed the survey, please be sure to click on the SUBMIT button; otherwise, your responses may not be captured. Your responses will be treated confidentially in accordance with data protection regulations. Your participation or failure to participate in this study will in no way affect your status as a student at ACOM. If you have any questions regarding the study, please contact the Co-Principal Investigators via email at Marinheirom@acom.edu or patelani@acom.edu. You can also contact the Primary Investigator, Dr. Benford Mafuvadze, at bmafuvadze@acom.edu. If you have any questions regarding your rights as a research subject, please contact the Review Board at IRB@acom.edu.

Part 1: Demographic data

How are you affiliated with ACOM?

OMS-I 

OMS-II

OMS-III

OMS-IV

Which of the following ethnicities do you identify as?

Caucasian/White

South Asian

East Asian or Native Hawaiian or Pacific Islander

African American or Black

Middle Eastern or North African

Hispanic or Latino

Other, please write in: 

Which religion do you currently practice? 

Christianity

Judiasm

Islam

Buddhism

Hinduism

Sikhism

Jainism

Atheism

Agnosticism

I do not belong to any faith tradition

Other (please write in): 

Which best describes your gender identity? 

Male

Female 

Non-binary

Questioning

Genderqueer

I do not wish to divulge this information 

Other (please write in): 

What best describes your sexual orientation? 

Heterosexual 

Bisexual

Gay

Lesbian

Queer

Questioning

I do not wish to divulge this information 

Are you aged? 

16-24 

25-34 

35-44 

45+ 

Do you currently have children 

Yes 

No 

Do you plan to have children 

During medical school (years 1-4) 

During residency

After residency 

No, I don’t have plans to have children 

Part 2: Knowledge of fertility and use of ART 

Overall, how would you rate your current knowledge of human-assisted reproductive technologies (ART) and fertility treatments 

No knowledge 

Some knowledge 

Fairly knowledgeable 

Very knowledgeable 

Taking birth control pills for more than 5 years negatively affects a woman’s fertility 

Definitely not 

Probably not 

Uncertain 

Probably 

Definitely 

Over 40 years, the success rate of fertility among women is around 50% 

Much lower 

Somewhat lower 

Do not know 

Somewhat higher 

Much higher 

The total cost of one cycle of in vitro fertilization is under $5,000 

Much lower 

Somewhat lower 

Do not know 

Somewhat higher 

Much higher 

There is a progressive decrease in a woman’s ability to become pregnant after the age of 35. 

Definitely not 

Probably not 

Uncertain 

Probably 

Definitely 

The rate of miscarriages is significantly higher for women in the 40s than for women in their 30s, even for physically fit women in excellent health. 

Definitely not 

Probably not 

Uncertain 

Probably 

Definitely 

Egg-freezing before the age of 35 can significantly prolong a woman’s fertility.

Definitely not 

Probably not 

Do not know 

Probably 

Definitely 

The age of her male partner is an important factor in a woman’s chances of becoming pregnant 

Definitely not 

Probably not 

Do not know 

Probably 

Definitely 

The use of in vitro fertilization poses some health risks for a woman. 

Definitely not 

Probably not 

Do not know 

Probably 

Definitely 

Children conceived using assisted reproductive technology (ART) have more long-term health problems than children conceived without the use of these fertility treatments.

Definitely not 

Probably not 

Do not know 

Probably 

Definitely 

Most fertility conditions are caused problems with the woman’s fertility 

Definitely not 

Probably not 

Do not know 

Probably 

Definitely 

Most women have to go through IVF more than once to have a baby 

Definitely not 

Probably not 

Do not know 

Probably 

Definitely 

The upper age limit for a man to be treated at most fertility clinics in the US is 55. 

Definitely not 

Probably not 

Do not know 

Probably 

Definitely 

Excessive alcohol consumption can increase infertility among both women and men. 

Definitely not 

Probably not 

Do not know 

Probably 

Definitely 

Frequent use of hot water baths and/or saunas can damage sperms. 

Definitely not 

Probably not 

Do not know 

Probably 

Definitely 

Part 3: Attitudes and other perspectives on ART and related fertility issues 

What do you think about the use of assisted reproductive technology in humans? 

Completely opposed 

Rather oppose 

Neither oppose nor support it 

Rather support 

Completely support 

Do you think that the availability of IVF and other ART encourages people to delay childbearing? 

Yes 

No 

Would you/have you ever considered using IVF treatment? Tick all that apply 

Yes, I/my partner have/has had IVF treatment 

Yes, I/my partner have/has considered IVF (but did not do it) 

Yes, I would definitely consider IVF treatment in the future, if I/my partner had fertility issues 

Yes, I (or wish that my partner) would consider IVF treatment in the future to start a family in later life (but we are not facing fertility issues now), e.g., social freezing 

No, I have never had, nor would I ever consider IVF

Do you support the practice of egg donation? 

Yes 

No 

Do you support the practice of sperm donation? 

Yes 

No 

Would you or your partner consider harvesting and freezing eggs to increase your chances of having your own biological children after the completion of your residency program? 

Yes 

No 

In what circumstances do you think IVF treatment should be funded by the federal government or by an individual/couple? 

When having a first child and unable to conceive naturally 

Federal government 

Privately 

In this circumstance, IVF should not be available 

When having another child (in addition to any they currently have) and unable to conceive naturally 

Federal government 

Privately 

In this circumstance, IVF should not be available 

When a decision has been made to have a child later in life due to medical studies and other national commitments such as military deployments 

Federal government 

Privately 

In this circumstance, IVF should not be available 

When fertility has been risked/sacrificed (e.g., due to cancer treatment) 

Federal government 

Privately 

In this circumstance, IVF should not be available 

What do you think should be the maximum number of rounds of IVF that the federal government should fund for any individual/couple? 

0 

1 

2 

3 

4 

5+ 

There should not be a maximum limit 

Why do you think there should be a maximum limit? Tick all that apply 

Because there are other ways to have a child (for example, adoption) 

Because there are better ways of allocating healthcare funds raised by taxpayers money (i.e., into other medical issues) 

Because of the potential psychological impact on the individual/couple 

Other, please specify 

How much would you pay to have a child through IVF? 

$0, would only do it if it is fully funded by the federal government 

$1-$2,500 

$2,600-$5,000 

$5,001-$10,000 

$10,001-$20,000 

$20,001-$25,000 

$25,001-$50,000 

>$50,001 

I will pay any amount to have my own child

Do you think that IVF should be available to single women without a partner (in this situation, sperm would be provided by a donor)? 

Yes 

No 

Do you think that IVF treatment should be available to same-sex couples (in this situation, sperm or egg would be provided by donors and surrogate mothers may be used)? 

Yes 

No 

Would you be comfortable with your partner being a sperm or egg donor? 

Yes 

No 

To what extent does your religion influence your views on the use of ART? 

Extensively 

Somewhat influence 

Neutral 

No influence at all 

Which of the following factors will likely influence your decision to use or not ever use ART? Tick all that applies. 

Cost 

Religion 

Residency program I will pursue 

Number of desired children 

Health status 

Fertility issues 

What do you think about the use of surrogate mothers by couples who are capable of carrying their own pregnancy? 

Completely opposed 

Rather oppose 

Neither oppose nor support it 

Rather support 

Completely support

In line with the recent Alabama Supreme Court ruling, do you think frozen embryos should be considered as “children” 

Definitely not 

Probably not 

Neutral 

Probably 

Definitely
